# Process optimization for enhancing production of *cis*-4-hydroxy-l-proline by engineered *Escherichia coli*

**DOI:** 10.1186/s12934-017-0821-7

**Published:** 2017-11-22

**Authors:** Kequan Chen, Yang Pang, Bowen Zhang, Jiao Feng, Sheng Xu, Xin Wang, Pingkai Ouyang

**Affiliations:** State Key Laboratory of Materials-Oriented Chemical Engineering, College of Biotechnology and Pharmaceutical Engineering, Nanjing Tech University, Nanjing, 211816 Jiangsu China

**Keywords:** *cis*-4-Hydroxy-l-proline, Process limitations, Two-strain coupling system

## Abstract

**Background:**

Understanding the bioprocess limitations is critical for the efficient design of biocatalysts to facilitate process feasibility and improve process economics. In this study, a proline hydroxylation process with recombinant *Escherichia coli* expressing l-proline *cis*-4-hydroxylase (SmP4H) was investigated. The factors that influencing the metabolism of microbial hosts and process economics were focused on for the optimization of *cis*-4-hydroxy-l-proline (CHOP) production.

**Results:**

In recombinant *E. coli*, SmP4H synthesis limitation was observed. After the optimization of expression system, CHOP production was improved in accordance with the enhanced SmP4H synthesis. Furthermore, the effects of the regulation of proline uptake and metabolism on whole-cell catalytic activity were investigated. The improved CHOP production by repressing *putA* gene responsible for l-proline degradation or overexpressing l-proline transporter *putP* on CHOP production suggested the important role of substrate uptake and metabolism on the whole-cell biocatalyst efficiency. Through genetically modifying these factors, the biocatalyst activity was significantly improved, and CHOP production was increased by twofold. Meanwhile, to further improve process economics, a two-strain coupling whole-cell system was established to supply co-substrate (α-ketoglutarate, α-KG) with a cheaper chemical l-glutamate as a starting material, and 13.5 g/L of CHOP was successfully produced.

**Conclusions:**

In this study, SmP4H expression, and l-proline uptake and degradation, were uncovered as the hurdles for microbial production of CHOP. Accordingly, the whole-cell biocatalysts were metabolically engineered for enhancing CHOP production. Meanwhile, a two-strain biotransformation system for CHOP biosynthesis was developed aiming at supplying α-KG more economically. Our work provided valuable insights into the design of recombinant microorganism to improve the biotransformation efficiency that catalyzed by Fe(II)/α-KG-dependent dioxygenase.

**Electronic supplementary material:**

The online version of this article (10.1186/s12934-017-0821-7) contains supplementary material, which is available to authorized users.

## Background

The microbial production of chemicals and materials has gained increasing attentions as the concerns on environmental problems and limited availability of fossil resource [1, 2]. More and more bioprocesses have been successfully developed to produce chemicals, fuels, or polymers with metabolically engineering microorganisms [3, 4]. Hydroxyprolines, such as *trans*-4-hydroxy-l-proline (THOP), and *cis*-4-hydroxy-l-proline (CHOP), are useful materials for pharmaceutical and cosmetic applications [5, 6]. Among them, CHOP could inhibit the collagen synthesis and its extracellular deposition as an analogue of l-proline, thereby reducing the growth of tumors [7]. In some bacteria, CHOP is directly produced via fermentation process and the related enzymes in CHOP synthetic pathway have been identified [8, 9]. Although the product titer is extremely low, these findings show the possibility of utilizing biological processes to produce CHOP.

**Fig. 1:**
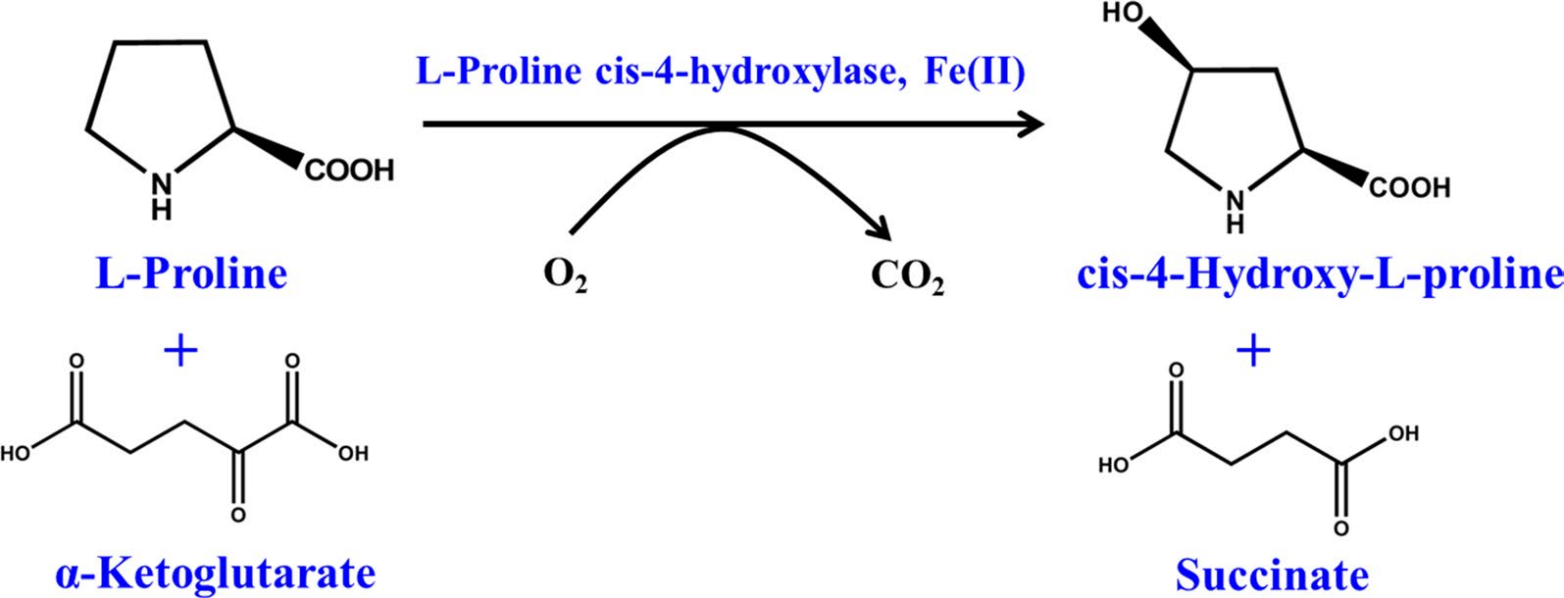
Scheme of *cis*-4-hydroxy-l-proline (CHOP) synthesis catalyzed by recombinant *E. coli* expressing l-proline *cis*-4-hydroxylase

Two l-proline *cis*-4-hydroxylases, which belong to the Fe(II)/2-oxoglutarate-dependent dioxygenase (Fe/αKG-DO) superfamily, were characterized from *Sinorhizobium meliloti* (SmP4H) and *Mesorhizobium loti* (MlP4H) [8]. These enzymes could catalyze the hydroxylation of l-proline at the *cis*-4-position to synthesize CHOP in the presence of α-ketoglutarate (α-KG), oxygen and ferrous ion (Fe^2+^) (Fig. 1) [8]. When *SmP4H* was expressed in *E. coli*, only 0.46 ± 0.15 µmol/g_wet cell weight_ CHOP was produced through the fermentation process [5]. Yi et al. reported that the availability of intracellular l-proline was strictly regulated to very low level in the wild type *E. coli* [10]. Meanwhile, the major generating pathway of α-KG, TCA cycle, was also tightly regulated during the l-proline hydroxylation process [10]. In addition, the compound CHOP is toxic and inhibits the growth of *E. coli* [5]. Thus, the efficient production of CHOP via direct fermentation is challenged by not only the complex metabolic engineering manipulation for the improving the supply of precursor l-proline and co-substrate α-KG, but also the low CHOP resistance.

The whole-cell biotransformation might be an alternative approach for CHOP bioproduction. Shibasaki et al. has already established the whole-cell process for the production of several hydroxyprolines, such as THOP and *cis*-3-hydroxy-l-proline [11]. However, the whole-cell process for CHOP production has never been investigated. When using whole-cell biocatalysts, numerous factors could potentially interrupt the catalytic performance, such as expression system, substrate uptake, co-substrate supply, substrate or product degradation by host endogenous metabolism, and product toxicity [10, 12]. The limiting factors for the effective CHOP bioproduction process remained largely uncharacterized.

In this study, we focused on investigating the factors that might influence the whole-cell biocatalyst efficiency for CHOP production (e.g., expression system, reaction condition, l-proline uptake, l-proline consumption by endogenous metabolism, and α-KG supply). Furthermore, the CHOP production process was optimized. Meanwhile, a two-strain coupling whole-cell biotransformation system was established for CHOP biosynthesis.

## Methods

### Bacterial strains and media

All bacterial strains used in this study are listed in Additional file 1: Table S1. Bacteria were grown in Luria–Bertani (LB) broth (10 g/L peptone, 5 g/L yeast extract and 5 g/L sodium chloride) or on LB agar (10 g/L peptone, 5 g/L yeast extract, 5 g/L sodium chloride and 10 g/L agar). Ampicillin (100 mg/L), chloramphenicol (15 mg/L), streptomycin (40 mg/L) or kanamycin (50 mg/L) was added when required.

### Plasmid construction

The plasmids constructed in this study were listed in Table 1. l-Proline *cis*-4-hydroxylase from *Sinorhizobium meliloti* NBRC 14782^T^ was codon-optimized for *E. coli* expression (http://www.jcat.de/) and synthesized by Genewiz (Suzhou, China). Detailed sequence information was provided in Additional file 1: Table S2. Gene *SmP4H* was subcloned into *Nde*I/*Xho*I sites of plasmid pACYCDuet-1, pCDFDuet-1, pRSFDuet-1, pET28a, pET22b and pETDuet-1 to give plasmid pACYCDuet-1-SmP4H, pCDFDuet-1-SmP4H, pRSFDuet-1-SmP4H, pET28a-SmP4H, pET22b-SmP4H and pETDuet-1-SmP4H respectively (Additional file 1: Figure S1). Gene *putP* was PCR amplified from *E. coli* BL21(DE3) genome and ligated to into plasmid pACYCDuet-1 to generate plasmid pACYCDuet-putP. Then the fragment of P_T7_:putP was PCR amplified with pACYCDuet-putP as template and ligated into *Xho*I site to give pET28a-SmP4H-putP. The l-glutamate oxidase (LOGX) from *Streptomyces ghanaensis* ATCC14672 was codon-optimized for *E. coli* expression and synthesized by Genewiz. The *Nde*I/*Xho*I-digested LOGX fragment was inserted to the *Nde*I/*Xho*I sites of pET28a to result in plasmid pET28a-LOGX.

**Table 1:** Plasmids used in this study

Plasmids	Description	Source
pACYCDuet-1	Expression vector, Cm^R^, P_T7_, P15A ori	Novagen
pCDFDuet-1	Expression vector, Sm^R^, P_T7_, CloDF13 ori	Novagen
pRSFDuet-1	Expression vector, Km^R^, P_T7_, RSF ori	Novagen
pET28a	Expression vector, Km^R^, P_T7_, pBR322 ori	Novagen
pET22b	Expression vector, Amp^R^, P_T7_, pBR322 ori	Novagen
pETDuet-1	Expression vector, Amp^R^, P_T7_, pBR322 ori	Novagen
pCDF303	Expression vector, Sm^R^, P_Trc_, pCDF ori	
pACYCDuet-1-SmP4H	Gene *SmP4H* inserted into *Nde*I/*Xho*I sites of pACYCDuet-1	This study
pCDFDuet-1-SmP4H	Gene *SmP4H* inserted into *Nde*I/*Xho*I sites of pCDFDuet-1	This study
pRSFDuet-1-SmP4H	Gene *SmP4H* inserted into *Nde*I/*Xho*I sites of pRSFDuet-1	This study
pET28a-SmP4H	Gene *SmP4H* inserted into *Nde*I/*Xho*I sites of pET28a	This study
pET22b-SmP4H	Gene *SmP4H* inserted into *Nde*I/*Xho*I sites of pET22b	This study
pETDuet-1-SmP4H	Gene *SmP4H* inserted into *Nde*I/*Xho*I sites of pETDuet-1	This study
pACYC-dCas9	Gene *dCas9* inserted into *Nco*I/*Avr*II sites of pACYCDuet-1	This study
pCDF-anti-putA	anti-putA (high) sgRNA sequences inserted into *Eco*RI/*Bam*HI sites of pCDF303	This study
pACYC-putP	Gene *putP* inserted into *Nco*I/*Xho*I sites of pACYCDuet-1	This study
pET28a-SmP4H-putP	Gene *putP* inserted into *Xho*I sites of pET28a-SmP4H	This study
pET28a-LOGX	Gene *LOGX* inserted into *Nde*I/*Xho*I sites of pET28a	This study

### Construction of CRISPRi system to repress gene putA

A catalytically dead Cas9 mutant (dCas9) derived from *Streptococcus pyogenes* [13] was synthesized by Genewiz (Suzhou, China) based on the pdCas9-bacterial plasmid (Addgene; Plasmid #44249), which contains a gene encoding dCas9 protein. *Nco*I/*Hin*dIII-digested dCas9 fragment was ligated into plasmid pACYCDuet-1. The sgRNA chimera, which contains four functional domains including a *Trc*-inducible promoter, a 20 bp complementary region for binding *putA* DNA, a 42 bp dCas9-binding hairpin and a 40 bp transcription terminator was synthesized by Genewiz (Nanjing, China) and inserted into *Eco*RI/*Bam*HI sites of pCDFDuet-1 to give plasmid pCDF-anti-putA (Additional file 1: Table S2). The plasmid pACYC-dCas9 and pCDF-anti-putA were co-transformed into *E. coli* BL21(DE3) to repress *putA* expression. PutA activity in crude extracts was determined by measuring P5C production (nmol P5C/min/mg protein) using o-aminobenzaldehyde as previously described [14].

### Cultivation of microorganisms

The recombinant strain was inoculated from a freshly transformed single colony on LB agar plate to 5 mL LB medium as seed culture. When cell growth reached stationary phase, 200 μL of seed culture was re-inoculated to 100 mL LB medium in a 250 mL flask. The cultures were then induced with 1.0 mM IPTG when OD_600_ reached 0.4–0.6 and allowed to grow for an additional 10 h at 30 °C and 200 rpm. The effects of induction OD_600_, temperature and IPTG concentration on the whole-cell activity were investigated.

### The biosynthesis of CHOP by whole-cell biotransformation

For the production of CHOP, the whole-cell biotransformation was carried out in a 50 mL flask with 20 mL reaction broth containing resting cells (OD_600_ = 10), 200 mM PBS buffer (pH = 6.5), 10 g/L l-proline, 13 g/L α-KG, 5.0 mM Fe^2+^, and 1.7 mM l-ascorbate. The reaction was performed at a temperature of 30 °C and stirred at 200 rpm. At the specific intervals of the reaction, samples were taken to measure the concentration of l-proline, α-KG and CHOP. To identify the factors limiting catalytic efficiency, the effects of surfactant solutions (0.5% Tween 80 and 0.5% Triton X-100), pH, Fe^2+^ concentration, and substrate concentration were investigated.

To supply α-KG economically, an engineered strain BL21/pET28a-LOGX that could convert l-glutamate to α-KG was coupled with CHOP producing strain. The two strains were collected, washed twice, and resuspended in a reaction mixture containing 200 mM PBS (pH 6.5), 10 g/L l-proline, 3.0 mM Fe^2+^, and 1.7 mM l-ascorbate. 5, 10 or 20 g/L of l-glutamate was supplemented to identify the suitable substrate ratio. In this coupling system, the final cell concentration was about 10 of OD_600_ for each strain. The reaction was carried out in a 50 mL flask with a work volume of 15 mL at 30 °C and 200 rpm. Samples were taken at the specific intervals to measure the concentration of CHOP, l-proline, l-glutamate, and α-KG.

### Analytical methods

Cell growth was monitored by measuring absorbance at 600 nm with BioMate 3S UV–visible spectrophotometer (Thermo). Aqueous concentrations of l-proline and CHOP were analyze by high-performance liquid chromatography (HPLC) system (Agilent 1100 series, Santa Clara, CA) equipped with a evaporative light scattering detector (ELSD) and a Prevail C18 column (250 × 4.6 mm, 5 µm, Bio-Rad, Hercules, CA, USA). The column temperature was maintained at 28.5 °C. The mobile phase was consisted of 0.7% (v/v) aqueous trifluoroacetic acid and 0.0653% (v/v) aqueous heptafluorobutyric acid and supplied at a flow rate of 1.0 mL/min. Analysis of α-KG and l-glutamate concentration was performed by a HPLC system (Agilent1290, Santa Clara, CA) equipped with the Agilent G1362A refractive index detector. The samples were separated on an Aminex HPX-87H ion-exchange column (Bio-Rad, Hercules, CA, USA) operating at 55 °C with 5 mM H_2_SO_4_ as the mobile phase (0.6 mL/min). The intracellular CHOP amount was analyzed by Waters time-of-flight mass spectrometry (GC–TOF–MS), which was equipped with a DB-5 fused-silica capillary column (30 m × 0.25 mm i.d., flm thickness 0.25 µm, J&W Scientifc, Folsom, CA). Quenching, metabolite extraction and derivatization were performed according to methods described previously [15]. The area of CHOP peak was then normalized to that of internal standard in the same chromatogram.

## Results

To develop an efficient whole-cell biotransformation system for microbial production of CHOP, it is critical to identify the key factors limiting whole-cell activity. Here, the factors including l-proline *cis*-4-hydroxylase expression, l-proline degradation by endogenous metabolism, l-proline uptake, and α-ketoglutarate (KG) supply were investigated.

### The expression of l-proline *cis*-4-hydroxylase in *E. coli*

**Fig. 2:**
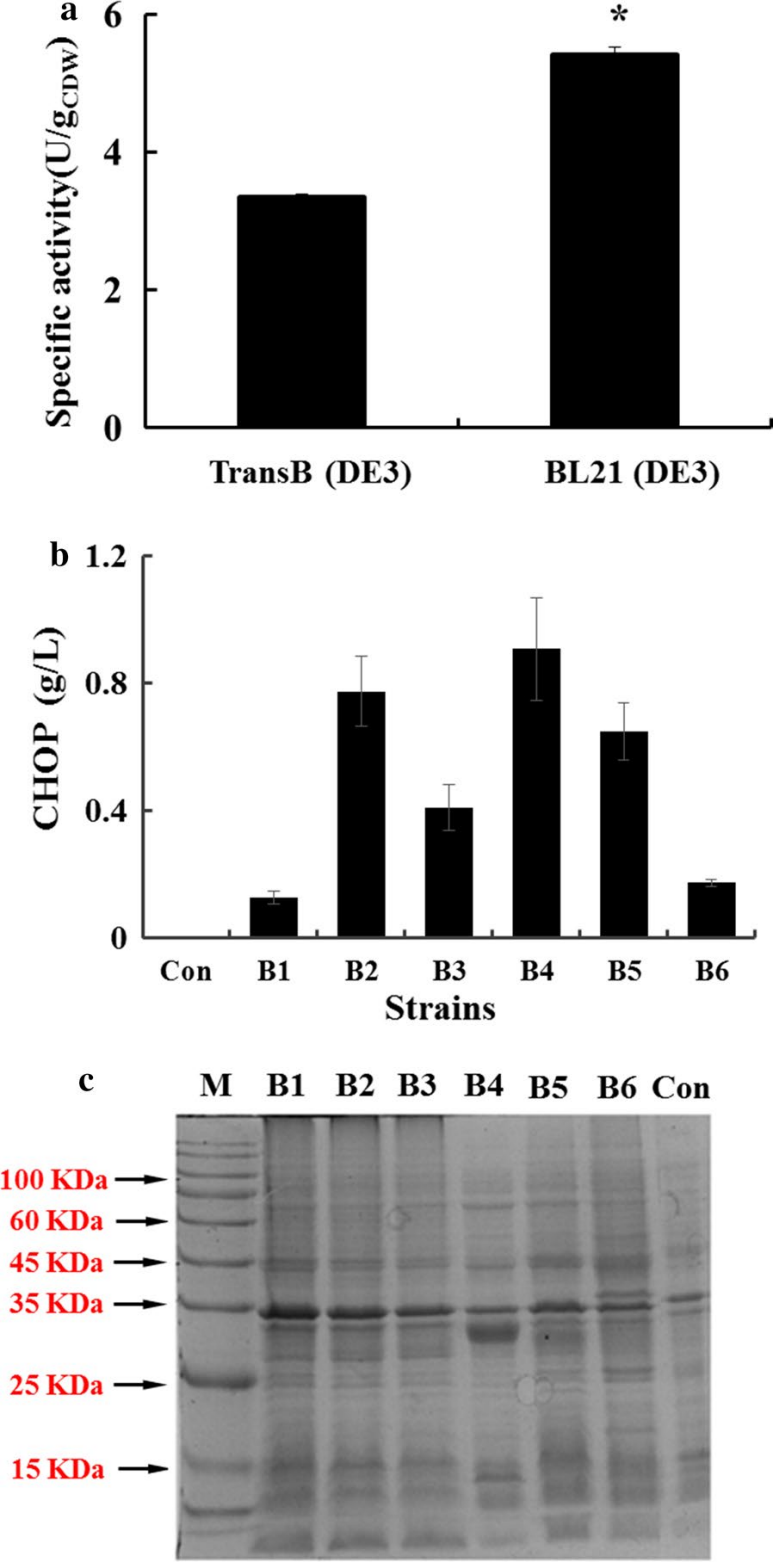
Optimizing the expression of l-proline *cis*-4-hydroxylase in *E. coli*. **a** CHOP production with different host cells. *SmP4H* was expressed in *E. coli* BL21(DE3) and TransB (DE3) respectively; **b** CHOP production from the resting cells of *E. coli* BL21(DE3) carrying different expression plasmids. **c** SDS-PAGE results for the soluble fractions of cell extracts of *E. coli* BL21(DE3) carrying different expression plasmids. Con, BL21(DE3); B1, BL21/pACYCDuet-1-SmP4H; B2, BL21/pCDFDuet-1-SmP4H; B3, BL21/pRSFDuet-SmP4H; B4, BL21/pET28a-SmP4H; B5, BL21/pET22b-SmP4H; B6, BL21/pETDuet-1 SmP4H

To obtain the whole-cell biocatalysts that could convert l-proline to CHOP, *SmP4H* from *S. meliloti* was cloned into plasmid pETDuet-1 and transformed into different *E. coli* host cells. As *E. coli* BL21/pETDuet-SmP4H exhibited higher catalytic activity than *E. coli* TransB/pETDuet-SmP4H (Fig. 2a), *E. coli* BL21(DE3) was selected as the host cell. However, obvious SmP4H synthesis was not observed in the recombinant *E. coli* BL21/pETDuet-SmP4H according to the SDS-PAGE analysis. Thus, the different expression systems were employed to optimize SmP4H synthesis (Additional file 1: Figure S1). The highest activity was observed in the resting cells of BL21/pET28a-SmP4H (Fig. 2b), which exhibited the highest SmP4H expression level by the SDS-PAGE analysis (Fig. 2c). These results indicated that the expression hosts and expression system had a significant effect on the whole-cell activity and thus influenced CHOP production.

The effects of cultivation conditions including induction temperature, IPTG concentration and induction OD_600_ on whole-cell activity of BL21/pET28a-SmP4H were also evaluated. Results showed that the decreasing induction temperature significantly increased whole-cell activity (Additional file 1: Figure S2). CHOP titer under condition of 20 °C was twofold higher than that under condition of 30 °C. Various IPTG concentrations ranging from 0.1 to 1 mM showed little effect on whole-cell activity. The optimal induction time was observed at the early exponential phase (Additional file 1: Figure S2).

### The effect of endogenous l-proline degradation on CHOP production

**Fig. 3:**
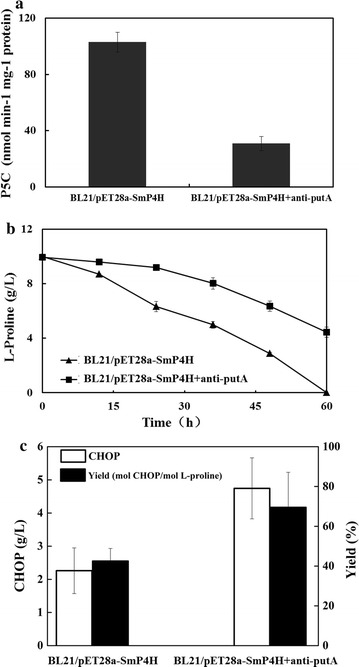
Improved CHOP production by repressing endogenous l-proline degradation with CRISPRi technology. **a** PutA activity in crude extracts of BL21/pET28a-SmP4H and BL21/pET28a-SmP4H+anti-putA; **b** the effect of *putA* repression on l-proline degradation in the whole-cell of BL21/SmP4H. The reaction mixture contained resting cells (OD_600_ = 10), 200 mM PBS buffer (pH = 6.5), 10 g/L l-proline; **c** the effect of *putA* repression on CHOP production in the whole-cell of BL21/SmP4H. The reaction mixture contained resting cells (OD_600_ = 10), 200 mM PBS buffer (pH = 6.5), 10 g/L l-proline, 13 g/L α-KG, 5.0 mM Fe^2+^, and 1.7 mM l-ascorbate

To evaluate whether l-proline degradation in *E. coli* BL21/pET28a-SmP4H affects the CHOP production, we employed the CRISPRi technology to repress the expression of *putA* gene (encoding l-proline dehydrogenase). To carry out the CRISPRi platform in *E. coli*, a heterologous gene coding for a red fluorescent protein was targeted by CRISPRi to confirm the successful utility of CRISPRi for gene repression (data not shown). When *putA* gene was targeted by CRISPRi, the PutA activity in the crude extracts of BL21/pET28a-SmP4H+anti-putA was reduced by 70% compared to that in BL21/pET28a-SmP4H (Fig. 3a). The l-proline degradation in the whole-cell of BL21/pET28a-SmP4H+anti-putA was also largely repressed as we expected (Fig. 3b). The CHOP production was increased to 3.9 g/L with a yield of 64.6% by repressing *putA* after bioconversion of 36 h, while only 2.3 g/L of CHOP was produced in the whole-cell of BL21/pET28a-SmP4H with a yield of 45.3% (Fig. 3c). These results indicated that the l-proline degradation by endogenous metabolism is one of the limited factors influencing CHOP production from l-proline.

### The effect of l-proline uptake on CHOP production

**Fig. 4:**
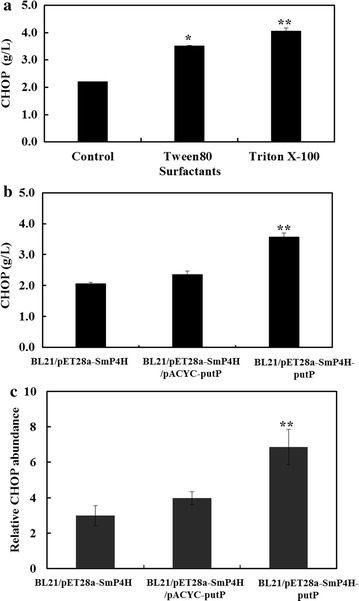
The effect of l-proline uptake on CHOP production. **a** The CHOP production by permeabilized cells of *E. coli* BL21/pET28a-SmP4H; **b** the effect of overexpressing *putP* in *E. coli* BL21/pET28a-SmP4H with different plasmids on CHOP production; **c** the effect of overexpressing *putP* in *E. coli* BL21/SmP4H with different plasmids on intracellular CHOP level. The relative CHOP abundance was calculated by normalizing the peak area of CHOP with internal standard. Results are expressed as mean ± standard error of the mean (n = 3). Significance levels of Students *t* test: *P < 0.01, **P < 0.001

In *E. coli*, l-proline is mainly transported into cells through a Na^+^/l-proline transporter encoded by gene *putP* [16]. To examine whether l-proline uptake over cellular membranes was limited and thus affected biocatalyst efficiency, cells of BL21/pET28a-SmP4H were permeabilized using Tween 80 or Triton X-100. Interestingly, the CHOP formation by permeabilized cells were all significantly higher than that by resting cells (Fig. 4a), suggesting that substrate uptake was limited during CHOP production. To address this problem, we modified the *putP* expression level in the strain BL21/pET28a-SmP4H. As shown in Fig. 4b, when gene *putP* was overexpressed with plasmid pACYCDuet-1, the CHOP production was improved to 2.4 from 2.0 g/L. With a higher copy number plasmid pET28a, the CHOP production was significantly improved, which could reach 3.6 g/L. Meanwhile, the intracellular CHOP accumulation was also evaluated. As the results illustrated in Fig. 4c, the higher level of intracellular CHOP was detected in the two *putP* overexpressing strains, while the recombinant strain BL21/pET28a-SmP4H-putP accumulated the highest level of intracellular CHOP. The effect of overexpressing *putP* on intracellular CHOP formation was well consistent with extracellular CHOP production. These results further reflected the l-proline uptake limitation in the whole-cell transformation system for CHOP production.

### The production of CHOP by the optimized whole-cell biocatalysts

**Fig. 5:**
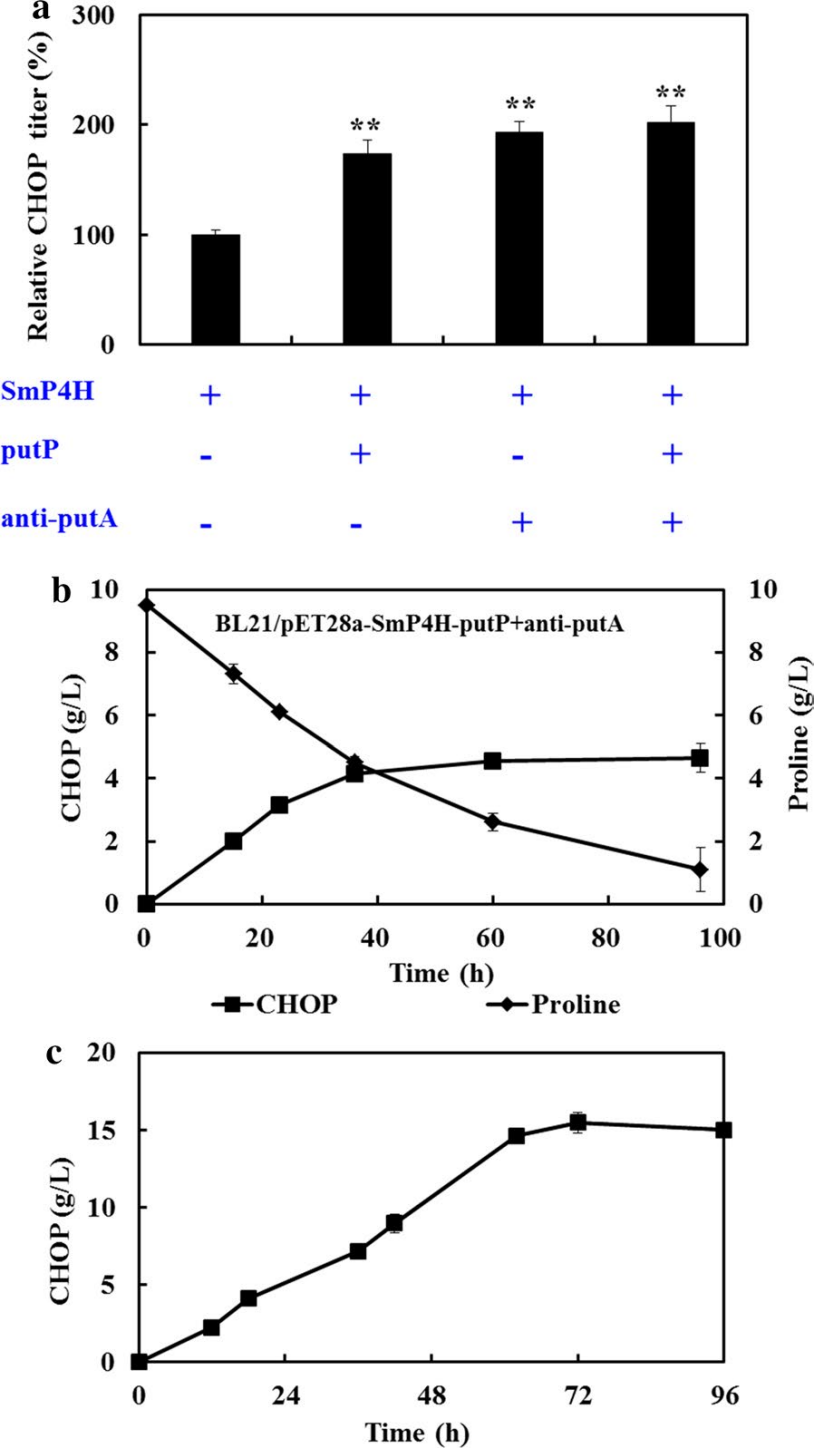
The CHOP production with the optimum recombinant *E. coli*. **a** The additive effect of repressing *putA* and overexpressing *putP* on CHOP production. Results are expressed as mean standard error of the mean (n = 3). Significance levels of Students t test: *P < 0.01, **P < 0.001; **b** the CHOP production and l-proline consumption by resting cells of *E. coli* BL21/pET28a-SmP4H-putP+anti-putA; **c** The synthesis of CHOP by the resting cells of BL21/pET28a-SmP4H-putP+anti-putA under the optimal reaction condition with a fed batch strategy

As mentioned above, weakened l-proline degradation and enhanced l-proline uptake could positively influence the whole-cell catalytic performance for CHOP production. To investigate if these two effects are additive, *E. coli* strain BL21/pET28a-SmP4H-putP+anti-putA was constructed by co-transformation of plasmid pET28a-SmP4H-putP, pACYC-dCas9, and pCDF-anti-putA. As the results illustrated in Fig. 5a, the final CHOP titer produced by the whole-cell of BL21/pET28a-SmP4H-putP and BL21/pET28a+anti-putA represented a 1.7- and 1.9-fold increase respectively compared with that by the whole-cell of BL21/pET28a-SmP4H. The highest CHOP production was observed when the strain BL21/pET28a-SmP4H-putP+anti-putA was used as whole-cell biocatalysts, which is 2.0-fold higher than that by the whole-cell of BL21/pET28a-SmP4H. It indicated that the beneficial effects of overexpressing putP and repressing putA on whole-cell activity were slightly additive.

Finally, the recombinant strain BL21/pET28a-SmP4H-putP+anti-putA was cultivated and collected to perform whole-cell biotransformation process for CHOP production. After bioconversion of 36 h, 4.2 g/L CHOP was produced from 10 g/L l-proline (Fig. 5b). The reaction conditions were also investigated. As shown in Additional file 1: Figure S3, the reaction pH significantly affected the whole-cell catalytic activity, which reached the highest level under the condition of pH 6.5. The concentration of Fe^2+^ had a minor effect. In addition, a substrate inhibition might exist in our whole-cell system when the l-proline concentration was above 10 g/L. With a fed-batch strategy, total CHOP accumulation reached a titer of approximately 15.6 g/L (Fig. 5c), which was the highest concentration reported thus far.

### The development of two-strain coupling whole-cell system to produce CHOP

**Fig. 6:**
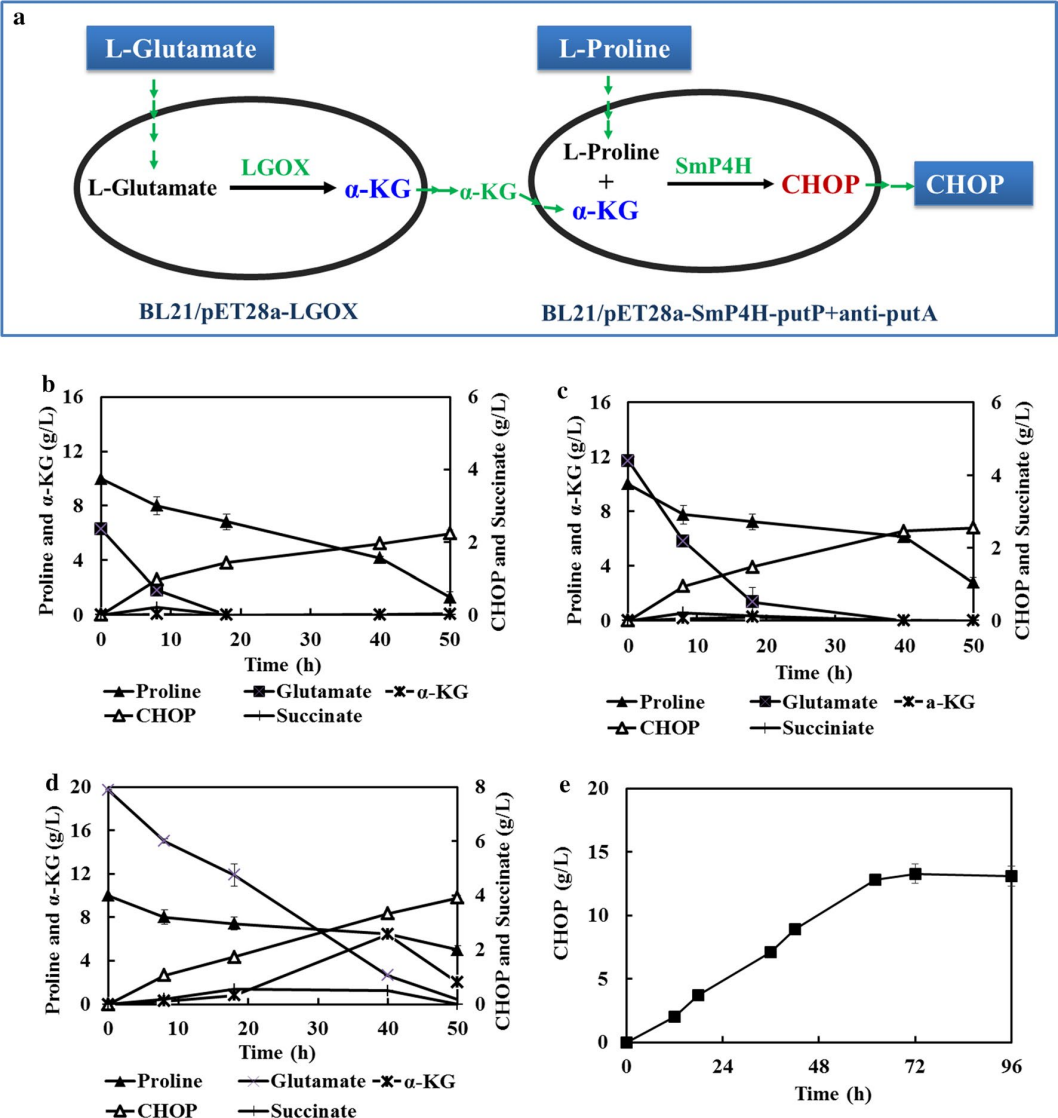
The two strain coupling whole-cell system to supply α-KG from l-glutamate to produce CHOP. **a** Scheme of CHOP synthesis catalyzed by the co-cultures of recombinant *E. coli* BL21/pET28a-LGOX and *E. coli* BL21/pET28a-SmP4H-putP+anti-putA; **b** the variance of CHOP, l-proline, l-glutamate and α-KG under the condition of 5 g/L l-glutamate and 10 g/L l-proline; **c** the variance of CHOP, l-proline, l-glutamate and α-KG under the condition of 10 g/L l-glutamate and 10 g/L l-proline; **d** the variance of CHOP, l-proline, l-glutamate and α-KG under the condition of 20 g/L l-glutamate and 10 g/L l-proline. **e** The CHOP production with a fed-batch strategy under the condition of 20 g/L l-glutamate and 10 g/L l-proline

In the whole-cell system for CHOP production, the extra supplement of α-KG was necessary as co-substrate. However, the high cost of α-KG might limit the potential for the industrial CHOP production. To address this problem, an engineered strain BL21/LGOX that expressed l-glutamate oxidase from *S. ghanaensis* was developed to synthetize α-KG from l-glutamate. This strain was then coupled with strain BL21/pET28a-SmP4H-putP+anti-putA as the co-catalysts for CHOP production (Fig. 6a). As we expected, the product CHOP was successfully detected. Then, varied concentration ratio of l-proline and l-glutamate was carried out to investigate their effects on CHOP production. Under the condition of 10 g/L l-proline, 2.2 g/L of CHOP was produced after the addition of 5 g/L l-glutamate (Fig. 6b), while the CHOP titer reached 2.5 g/L when l-glutamate concentration was increased to 10 g/L (Fig. 6c). When the two-strain coupling system was supplemented with 10 g/L l-proline and 20 g/L l-glutamate, the CHOP production could be improved to 3.9 g/L. Meanwhile, the accumulation of α-KG was observed (Fig. 6d). Under this condition, 13.5 g/L of CHOP could be accumulated with a fed-batch strategy (Fig. 6e). The titer was similar with that produced by the single strain system, indicating the potential application of our two-strain coupling system for the production of l-proline hydroxylase derived compounds. In addition, we found that a negligible amount of succinate as detected in the whole-cell system (Fig. 6), probably due to the endogenous degradation.

## Discussion

Hydroxy amino acids are important intermediates in the industrial synthesis of valuable chiral compounds [17–19]. For their microbial production, several free amino acid-hydroxylating enzymes have been characterized [20]. In our current work, to develop an efficient whole-cell process for CHOP production, the recombinant *E. coli* expressing l-proline *cis*-3-hydroxylase was constructed, and factors associated with microbial physiology were focused on to identify the process limitations that impact the efficiency of biocatalytic transformation.

Initially, to explore the expression system suitable for SmP4H expression, two different *E. coli* host strains and six expression plasmids were employed. The engineered strain BL21/pET28a-SmP4H, which showed highest SmP4H expression level, exhibited the highest biocatalytic activity, while SmP4H synthesis in other system was poor (Fig. 2). These results suggested a SmP4H synthesis limitation in *E. coli*. Several other microbial physiology related factors such as substrate uptake and degradation were then investigated. To reduce the l-proline degradation by host intrinsic enzymes, the expression of gene *putA* in BL21/pET28a-SmP4H was repressed using a CRISPRi technology. Its beneficial effect on CHOP production and yield suggested that l-proline hydroxylation process was limited by intracellular l-proline availability (Fig. 3). In addition, substrate transporters have been reported as excellent targets for strain improvement in the whole-cell system [21]. By using permeabilized cells, the improved biocatalytic activity inferred the limitations of the l-proline uptake. When a Na^+^/l-proline permease putP was overexpressed in the SmP4H containing strain BL21/pET28a-SmP4H, the intracellular and extracellular CHOP accumulation were all positively affected correlated with the increased *putP* expression level. These results further indicate that factors affecting intracellular l-proline availability were critical for improving biocatalysts activity in the whole-cell bioconversion system for CHOP production, which was consistent with the conclusion from the previous studies that l-proline uptake and competition of THOP formation from l-proline by l-proline-4-hydroxylase with l-proline catabolism were the key factors limiting biocatalytic efficiency for THOP production [22]. However, the additive effect of overexpressing *putP* and repressing *putA* was slight (Fig. 5). Increasing the extracellular l-proline concentration could not substantially enhance CHOP production. These results suggested that engineering strategies aiming at further improving SmP4H synthesis and catalytic properties might be promising for CHOP biosynthesis.

For the biocatalytic process that utilizes *α*-KG dependent dioxygenases, α-KG was required as a co-substrate. The efficiency of α-KG dependent biotransformation in resting cells has been reported to be limited by the *α*-KG supply in the previous study [10, 22, 23]. In our work, the exogenous supply of α-KG was carried out as an alternative method to solve this problem. However, it was a cost burden to the bioconversion process. Thus, the solution of α-KG supply problem has important significance for developing an economically viable process. The major strategy that has been already employed was to reconstitute the tricarboxylic acid (TCA) cycle of *E. coli* to force and increase α-KG flux through α-KG dependent hydroxylase [23, 24]. However, the α-KG production via these approaches remained far below that required for industrial applications. l-Glutamate was thought as a promising starting material for α-KG production since it was an industry at overcapacity [25]. Along this line of consideration, we developed a novel strategy to relieve α-KG supply limitation during CHOP production process with a two-strain coupling system, where one of the strains expressing LGOX was constructed to supply α-KG from l-glutamate. Excitingly, CHOP was successfully synthesized in our two-strain coupling system (Fig. 6). At a lower level of l-glutamate concentration, the CHOP titer was also limited by the lack of α-KG, probably due to the degradation of α-KG by endogenous metabolism (Additional file 1: Figure S4). With excessive l-glutamate supplementation, the CHOP titer could reach 3.9 g/L, a similar level with the extra addition of α-KG. To improve the efficiency of this system, engineering efforts to reduce α-KG consumption would be focused on in the future studies.

Our work clearly demonstrated the factors that could influence biocatalytic performance in a whole-cell bioconversion system for CHOP production. Through artificially engineering host phenotypes, the biocatalysts efficiency has been largely improved. Meanwhile, with a two-strain coupling system, the whole-cell process was more economical. Finally, 13.5 g/L of CHOP, the highest titer reported so far, was obtained in our work. To our knowledge, this is the first report that *E. coli* strain was artificially engineered to product CHOP with a whole-cell biotransformation process, especially with a two-strain coupling whole-cell process. However, this titer was remained far below that required for industrialization application. To further investigate this issue, the capacity of the cell needs to be utilized efficiently by optimized l-proline *cis*-4-hydroxylase expression with some engineering approaches, i.e., using “trial and error” approaches and modifying the structure of the 5′end of the mRNA [26, 27]. In addition, the host strain will be further metabolically engineered to modify characteristics regarding l-proline uptake and α-KG consumption.

## Conclusions

Identifying limitations for the whole-cell SmP4H-catalyzed process was demonstrated as the first step toward process development for CHOP production. A strong interference of microbial catalytic activity with the SmP4H expression, and l-proline uptake and metabolism was demonstrated in our study. Taken together, we have established some engineering strategies to address these limitations by optimizing the expression system, repressing *putA* gene and overexpressing a l-proline transporter putP. Meanwhile, to reduce the material cost and make the process more economically feasible, a two-strain coupling whole-cell system was developed to address the α-KG supply problem. This work highlights the limitations for Fe/αKG-DO dependent process and provided opportunities for further engineering of recombinant biocatalysts to facilitate process feasibility and improve process economics.

## Additional file

**Additional file 1.** Additional tables and figures.
