# Supplementary material for: Process optimization for enhancing production of cis-4-hydroxy-l-proline by engineered Escherichia coli
Source: Microb Cell Fact. 2017 Nov 22;16:210. doi: 10.1186/s12934-017-0821-7 (PMC5700529; doi:10.1186/s12934-017-0821-7)
Supplement: Supplementary file 1 — Additional file 1. Additional tables and figures. [file 12934_2017_821_MOESM1_ESM.pdf]

Applied Microbiology and Biotechnology

Process optimization for enhancing production of cis-4-hydroxy-l-proline by engineered *Escherichia coli*

Kequan Chen, Yang Pang, Bowen Zhang, Jiao Feng, Sheng Xu, Xin Wang*, Pingkai Ouyang

State Key Laboratory of Materials-Oriented Chemical Engineering, College of Biotechnology and Pharmaceutical Engineering, Nanjing Tech University, Nanjing 211816, Jiangsu, China

*Corresponding authors: Xin Wang

Phone: +86-186-518-20191

E-mail: xinwang1988@njtech.edu.cn

Table S1 Strains used in this study

Strains	Description	Source
E. coli Trans T1	Used for general gene cloning	TransGen
E. coli BL21(DE3)	Used as host strain	TransGen
E. coli TranB(DE3)	Used as host strain	TransGen
BL21/pACYCDuet-SmP4H	*E. coli* BL21(DE3) harboring plasmid pACYCDuet-1-SmP4H	This study
BL21/pCDFDuet-SmP4H	*E. coli* BL21(DE3) harboring plasmid pCDFDuet-1-SmP4H	This study
BL21/pRSFDuet-SmP4H	*E. coli* BL21(DE3) harboring plasmid pRSFDuet-1-SmP4H	This study
BL21/pET28a-SmP4H	*E. coli* BL21(DE3) harboring plasmid pET28a-SmP4H	This study
BL21/pET22b-SmP4H	*E. coli* BL21(DE3) harboring plasmid pET22b-SmP4H	This study
BL21/pETDuet-SmP4H	*E. coli* BL21(DE3) harboring plasmid pETDuet-1-SmP4H	This study
TransB/pETDuet-SmP4H	*E. coli* BL21(DE3) harboring plasmid pETDuet-1-SmP4H	This study
BL21/pET28a+anti-putA	*E. coli* BL21(DE3) harboring plasmid pET28a, pACYC-dCas9, and pCDF-anti-putA	This study
BL21/pET28a-SmP4H+anti-putA	*E. coli* BL21(DE3) harboring plasmid pET28a-SmP4H, pACYC-dCas9, and pCDF-anti-putA	This study
BL21/pET28a-SmP4H /pACYC-putP	*E. coli* BL21(DE3) harboring plasmid pET28a-SmP4H and pACYC-putP	This study
BL21/pET28a-SmP4H-putP	*E. coli* BL21(DE3) harboring plasmid pET28a-SmP4H-putP	This study
BL21/pET28a-SmP4H-putP+anti-putA	*E. coli* BL21(DE3) harboring plasmid pET28a-SmP4H-putP, pACYC-dCas9, and pCDF-anti-putA	This study

Table S2. Codon-optimized synthetic gene in this study

**Name**	**Sequence（5′ >3′）**
**SmP4H**	ATGAGCACCCACTTCCTGGGCAAGGTGAAATTTGACGAAGCCCGTCTGGCGGAAGATC<br>TGAGCACCCTGGAAGTGGCGGAATTTAGCAGCGCCTACAGCGATTTTGCCTGCGGCAA<br>GTGGGAAGCGTGTGTGCTGCGTAACCGCACGGGCATGCAGGAAGAGGATATCGTGGTG<br>AGCCATAATGCGCCGGCGCTGGCCACCCCGTTAAGCAAAAGCCTGCCGTACCTGAACG<br>AACTGGTGGAAACCCACTTCGATTGCAGCGCGGTGCGCTATACCCGCATTGTGCGCGTG<br>AGCGAAAACGCGTGCATCATTCCGCACAGCGACTATCTGGAACTGGACGAAACCTTCA<br>CCCGCCTGCACCTGGTTTTAGACACCAATAGCGGCTGCGCGAACACGGAGGAAGACAA<br>AATCTTCCACATGGGCCTGGGCGAAATCTGGTTTCTGGACGCGATGCTGCCGCACAGCG<br>CGGCCTGTTTTAGCAAAACCCCGCGTCTGCACCTGATGATCGATTTTGAGGCCACGGCG<br>TTTCCGGAAAGCTTTCTGCGCAACGTTGAACAGCCGGTGACCACCCGCGATATGGTTGA<br>TCCTCGCAAAGAACTGACGGACGAAGTGATCGAAGGCATCCTGGGCTTCAGCATCATCA<br>TCAGCGAAGCCAACTACCGCGAGATCGTGAGCATTCTGGCGAAGCTGCACTTCTTCTAC<br>AAAGCGGATTGCCGCAGCATGTACGATTGGTTAAAGGAAATTTGCAAACGCCGCGGCGA<br>TCCGGCGCTGATTGAAAAAACCGCGAGCCTGGAGCGCTTCTTTCTGGGCCATCGCGAA<br>CGCGGCGAGGTGATGACCTATTAA
**SgRNA for anti-putA**	GTCGTCCAGCTTAACCCCCAGTTTTAGAGCTAGAAATAGCAAGTTAAAATAAGGCTAGT<br>CCGTTATCAACTTGAAAAAGTGGCACCGAGTCGGTGCTTTTTTT

Figure S1 The schematic of expressing SmP4H with different expression plasmids in *E. coli*.

Figure S2 The effects of cultivation conditions including induction temperature, IPTG concentration and induction OD_{600} on whole-cell activity of BL21/ pET28a-SmP4H.

Figure S3 The effects of reaction conditions including pH, l-proline concentration and Fe^{2+} concentration on CHOP production.

Figure S4 The synthesis of CHOP by the resting cells of BL21/pET28a-SmP4H-putP+anti-putA under the optimal reaction condition with a fed batch strategy.

Figure S5 The α-KG production by the resting cells of BL21/pET28a-LGOX when l-glutamate was supplemented at a concentration of 5 g/L, 10 g/L and 20 g/L respectively.

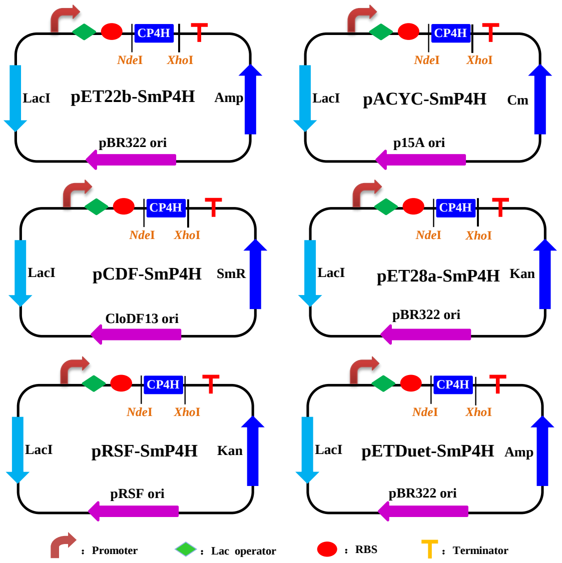

Figure S1 (Chen et al., Process optimization for……)

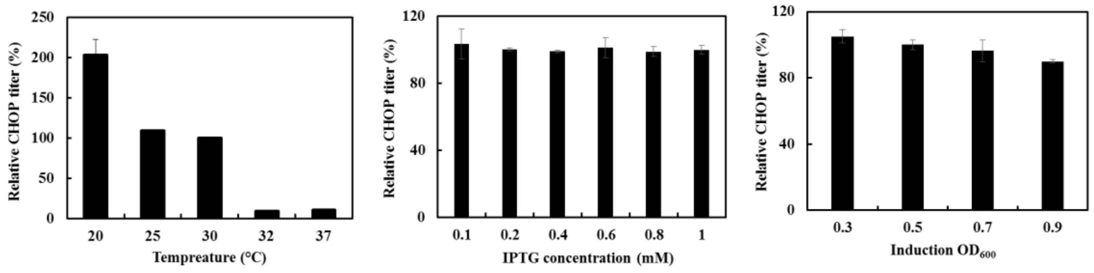

Figure S2 (Chen et al., Process optimization for……)

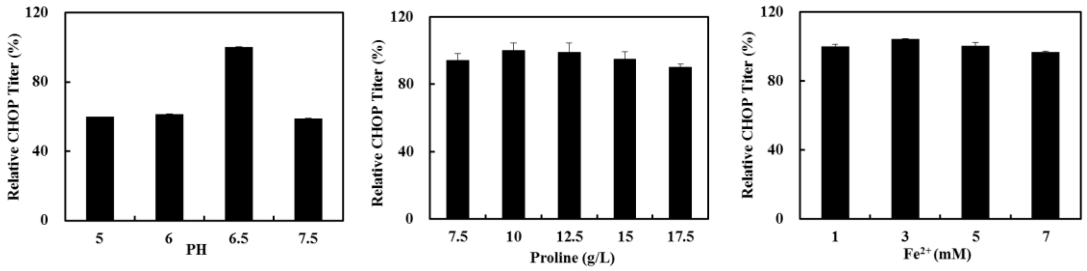

Figure S3 (Chen et al., Process optimization for……)

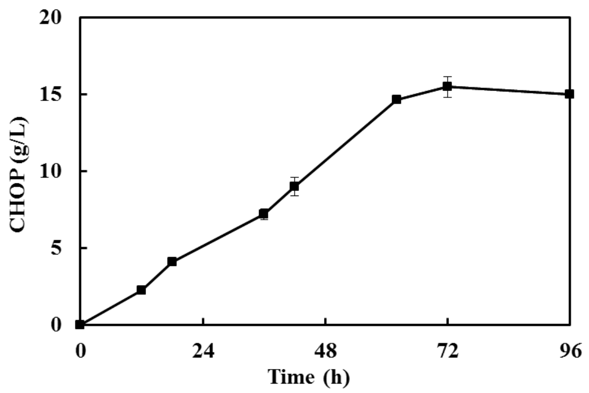

Figure S4 (Chen et al., Process optimization for……)

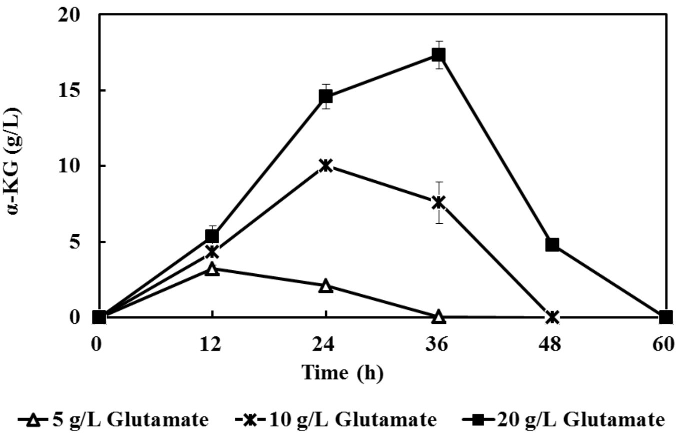

Figure S5 (Chen et al., Process optimization for……)
